# Isolation, Expansion and Transplantation of Postnatal Murine Progenitor Cells of the Enteric Nervous System

**DOI:** 10.1371/journal.pone.0097792

**Published:** 2014-05-28

**Authors:** Heike Monika Dettmann, Ying Zhang, Nadine Wronna, Udo Kraushaar, Elke Guenther, Roland Mohr, Peter Helmut Neckel, Andreas Mack, Joerg Fuchs, Lothar Just, Florian Obermayr

**Affiliations:** 1 Department of Pediatric Surgery and Pediatric Urology, University Children's Hospital Tuebingen, Tuebingen, Germany; 2 Institute of Anatomy, Center for Regenerative Biology and Medicine, University of Tuebingen, Tuebingen, Germany; 3 Deptartment of Electrophysiology, Natural and Medical Sciences Institute at the University Tuebingen, Reutlingen, Germany; Temple University School of Medicine, United States of America

## Abstract

Neural stem or progenitor cells have been proposed to restore gastrointestinal function in patients suffering from congenital or acquired defects of the enteric nervous system. Various, mainly embryonic cell sources have been identified for this purpose. However, immunological and ethical issues make a postnatal cell based therapy desirable. We therefore evaluated and quantified the potential of progenitor cells of the postnatal murine enteric nervous system to give rise to neurons and glial cells *in vitro*. Electrophysiological analysis and BrdU uptake studies provided direct evidence that generated neurons derive from expanded cells *in vitro*. Transplantation of isolated and expanded postnatal progenitor cells into the distal colon of adult mice demonstrated cell survival for 12 weeks (end of study). Implanted cells migrated within the gut wall and differentiated into neurons and glial cells, both of which were shown to derive from proliferated cells by BrdU uptake. This study indicates that progenitor cells isolated from the postnatal enteric nervous system might have the potential to serve as a source for a cell based therapy for neurogastrointestinal motility disorders. However, further studies are necessary to provide evidence that the generated cells are capable to positively influence the motility of the diseased gastrointestinal tract.

## Introduction

The enteric nervous system (ENS) derives from vagal and sacral progenitor cells of the neural crest [1]. Neural crest stem cells enter the proximal foregut via the dorsal aorta by embryonic day 9.5 in mice. Driven by a complex program of genes [2], [3] the cells proliferate and migrate in a rostro-to-caudal direction in order to colonize the entire gut by embryonic day 14.5. After colonization the cells generate a network of neuronal and glial cells providing a complex circuitry that coordinates the motility of the gastrointestinal tract and is involved in regulation of its secretory activity, blood flow and modulation of the immune system [4]–[8].

Impairment of ENS development leads to dys- or aganglionosis of the intestine, resulting in peristaltic dysregulation, intestinal obstruction and enterocolitis as in Hirschsprung's Disease [9]–[11]. Apart from congenital disturbances, age and disease related neurodegenerative changes of the bowel are of increasing clinical interest taking demographic developments into account [12]–[14]. Therapeutic options are limited for both, congenital and acquired neural enteropathies. Surgery for Hirschsprung's disease, although life-saving, frequently results in impaired bowel function and reduced quality of life [15]. Treatment strategies for age and disease related enteric neuropathies are even less defined and mainly palliative.

Neural stem or progenitor cells have been proposed to restore gastrointestinal function in patients suffering from enteric neuropathies [16]–[18]. Candidate cells for this purpose have been identified and isolated from embryonic, postnatal and adult gut of rodents [19]–[22]. In addition progenitor cells of the human postnatal ENS could be identified by several groups [23]–[27].

*In vivo* grafting experiments demonstrated integration of rodent embryonic CNS and ENS progenitor cells into rodent gut and indicate their positive impact on gastrointestinal function in several animal models [28]. Since neurogastrointestinal motility disorders are diagnosed after birth, however, the application of pluripotent or fetal multipotent stem cells is associated with safety, practical and ethical problems. To meet requirements of a clinically relevant scenario, autologous progenitor cells need to be isolated postnatally from a readily accessible cell source [25].

However, little is known about the potential of postnatal ENS progenitor cells to give rise to electrically active neurons and their ability to generate neurons and glial cells, when implanted into postnatal or adult gut *in vivo* [29]–[32]. In the present study, murine postnatal ENS progenitors were expanded *in vitro* and grafted into the distal colonic wall of immunocompromised mice to investigate cellular integration into the intestinal microenvironment *in vivo*. We demonstrate long-term survival, migration and differentiation of *in vitro* generated cells in the recipient gut, confirming data of the previous work of Hotta et al. [32]. In order to verify the cell biological characteristics of grafted cells, BrdU proliferation assay, immunocytochemistry, and electrophysiological patch clamp analysis were performed on proliferating and differentiated neural progenitors derived from postnatal intestine.

## Materials and Methods

### Animals

Animal experiments were approved by the local Committee on Use and Care of animals at the University of Tuebingen. Neonatal (P0) intestinal tissue was obtained from C57BL/6 and eGFP transgenic mice expressing an actin-eGFP reporter gene. eGFP transgenic mice were kindly provided by Dr. M. Okabe, Osaka University, Japan. Mice ubiquitously expressing eGFP were used to enable identification of donor derived cells after implantation into the recipient gut. Adult immunodeficient NOD.Cg-Prkdc^scid^ IL2rg^tm1WJl^ (Charles River, Sulzfeld, Germany) were used as host for neurosphere implantation studies.

### Neurosphere preparation and cell culture

The entire gut of the pups (P0–P4) was removed, longitudinal and circular muscle layers were dissected and finely diced. The tissue was incubated in collagenase (750 U/mL; Sigma, Frickenhausen, Germany) and dispase (250 µg/mL; Roche, Mannheim, Germany) dissolved in Hank's buffered salt solution (HBSS) with Ca^2+^ and Mg^2+^ (PAA, Pasching, Austria) for 30 min at 37°C. After 10 min 0.05% DNase I (Sigma) was added. At the end of digestion the tissue was triturated with a fire-polished blue tip and fetal calf serum was added (final concentration, 10%). Cell suspension was washed once in HBSS without Ca^2+^ and Mg^2+^ by centrifugation at 200 *g* for 6 min at room temperature. After another washing step with DMEM/F-12 the cell pellet was re-suspended in DMEM/F-12 medium supplemented with N2 (1∶100; Invitrogen), basic fibroblast growth factor (bFGF, 20 ng/mL, Sigma), EGF (20 ng/mL; Sigma), penicillin/streptomycin 100× (1∶100; PAA) and L-glutamine 200 mM (1∶100; PAA). Dissociated cells were seeded into six-well culture plates (2.5×10^4^ cells per well). On the first day of cultivation B27 (1∶50; Gibco, Karlsruhe, Germany) was supplemented. The culture medium was changed every 3 days, growth factors were freshly added daily. Cells were cultured in a humidified incubator at 37°C and 5% CO_2_. For cell differentiation, neurospheres were seeded on 48 well cell culture plates coated with Laminin (1.5 µg/mL, Sigma), Fibronectin (10 µg/mL, Sigma), Poly-L-Ornithin (1 µg/mL, Sigma) or glass cover slips coated with 5 µg/cm^2^ rat tail collagen type I (BP Bioscience) and cultured up to 8 weeks in culture medium (DMEM/F-12 medium supplemented with N2, penicillin/streptomycin, L-glutamine, ascorbate-2-phosphate (200 µmol/L, Sigma), and 2% fetal calf serum (PAA)).

### Growth and long-term expansion of enteric neurospheres

To evaluate the growth of the neurospheres, we measured size and number of spheroids larger than 20 µm in diameter after one and after 5 days *in vitro*.

In order to demonstrate long-term expansion of isolated cells while maintaining their proliferation and differentiation capacity, secondary and tertiary enteric neurospheres were generated. Isolated cells were cultivated as mentioned above. After 10 and 20 days of culture generated spheres were chopped in to small pieces (100 µm, McIlwaen Tissue Chopper, Mickle Laboratory Engineering Co. Ltd, Guildford, UK). Tertiary spheres were exposed to BrdU at day 25 and proliferated for another 3 days before differentiated for another 10 days and processed immunohistochemically as described below.

### Immunohistochemistry

Immunohistochemistry was performed on paraformaldehyde (4%) fixed cell cultures, paraffin sections (5 µm) and cryosections (10 µm) as described elsewhere [33]. The following primary antibodies were used: rabbit anti-Neural Class (III) beta-Tubulin (1∶2,000; TUJ1, Covance, Münster, Germany), rabbit anti-Nitric Oxide Synthase (1∶5,000; R&D, Las Vegas, USA), rabbit anti-protein gene product 9.5 (1∶800; AbD Serotec, Oxford, United Kingdom), rabbit anti-glial fibrillary acidic protein (1∶500; Dako, Glostrup, Danmark), rabbit anti-S100β (1∶200; abcam, Cambridge, Great Britain), mouse-anti human neuronal protein Hu c/d (1∶50; Invitrogen), chicken anti-green fluorescent protein (1∶500; abcam) and rat-anti BrdU (1∶50; AbD Serotec). Detection of the primary antibodies was performed with the following fluorochrome-linked secondary antibodies: goat anti-rabbit IgG Cy3 (1∶400; Invitrogen), goat anti-rat Cy3 (1∶400; Invitrogen), goat anti-rabbit IgG Cy5 (1∶500; Invitrogen) and goat anti-chicken IgY, Dylight 488 (1∶500; abcam). For identifying biocytin filled cells Cy^3^-conjugated streptavidin antibody (1∶500, Jackson ImmunoResearch, United Kingdom) was used.

Cell nuclei were stained with 4′-6-diamidino-2-phenylinodole (DAPI) solution (200 ng/mL; Invitrogen).

### Electrophysiology

Expanded neurospheres, generated from postnatal murine intestine were seeded on collagen type I coated glass cover slips and kept under differentiation conditions for up to 36 days. For these studies culture medium was supplemented with GDNF (10 ng/mL, Sigma). The electrodes had a resistance between 1 and 6 MΩ; the intracellular solution consisted of (in mM) KCl 130, ethylene glycol- bis(β-aminoethyl ether) N, N, N′, N′ – tetraacetic acid 10 for voltage clamp and 0.1 for current clamp experiments, MgCl_2_ 2, K_2_-ATP 2, HEPES 10, pH adjusted to 7.2 with KOH, and the extracellular solution consisted of (in mM) NaCl 135, KCl 2.5, MgCl_2_ 1, CaCl_2_ 2, 1, N-(2- hydroxyethyl) piperazine-N′-(2-ethanesulphonic acid (HEPES) 10, Glucose 10, pH adjusted to 7.35 with NaOH. In some of the experiments biocytin (0.2%; Tocris Biosciences, USA) was added to the intracellular solution. Cells were held in voltage clamp and current clamp mode, respectively, by an EPC10 amplifier (HEKA, Germany), and currents or voltages were filtered at 2.9 kHz using the internal Bessel filter, digitized and stored at 50 kHz. Cells were held at −70 mV between the different recordings. Experiments were analyzed using PAT (J. Bergsman) and self-written macros in Igor Pro 6.22 (Wavemetrics, USA). For voltage clamp experiments 250 ms lasting voltage steps from −120 mV to +40 mV in 10 mV increments were preceded by a conditioning voltage step (−120 mV, 100 ms). The time interval between the steps was 5 s. For current clamp experiments cells were adjusted to a membrane voltage of approximately −70 mV and current pulses between −200 and +1000 pA (100 pA increment) were applied for 150 ms. The time interval between the sweeps was 10 s.

The activation and inactivation curves were obtained from the peak current-voltage relation and currents were converted into conductance using
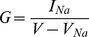
with *G* being the conductance, *I_Na_* the peak current, *V* the applied voltage step and *V_Na_* being the reversal potential of the Na^+^ current according to Nernst. The curves were fitted with simple Boltzmann functions,
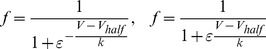
for activation and inactivation, respectively, where *V* is the membrane potential, *V_half_* is the potential at which the value of the Boltzmann function is 0.5, and *k* is the slope factor. Data values denote mean ± standard error of the mean (SEM) unless stated differently.

### In vivo cell implantation

Cells for *in vivo* implantation studies were generated from neonatal (P0–4) gut of eGFP transgenic C57BL/6 mice. Neurospheres were formed by proliferating cells for 7 days *in vitro* without induction of differentiation. Eight weeks old NOD.Cg-Prkdc^scid^ IL2rg^tm1WJl^ mice (25–30 g) were anesthesized with ketamine (100 mg/kg) and xylazine (5 mg/kg) intraperitoneally. A midline abdominal incision was performed. Neurospheres (100 µl; 200 neurospheres/mL) were injected into the distal colonic wall using a 30 gauge needle at two distinct sites. All implantation experiments were performed using a binocular microscope (Olympus SD 30, Olympus, Hamburg, Germany). The mice had free access to food and water postoperatively. 3 and 12 weeks after implantation animals were sacrificed and grafted guts were removed for immunohistological analysis.

### BrdU proliferation assay

To evaluate whether *in vitro* generated, implanted or electrophysiologically recorded cells had proliferated *in vitro*, postnatal murine ENS precursor cell cultures were supplemented with BrdU (1 µM) at day 1 after cell isolation. Cells were proliferated for 7 days and then washed twice with sterile PBS (Biochrom) to remove BrdU from the cell suspension before differentiation *in vitro* or implantation into the distal mouse colon. Paraformaldehyde fixed cryosections from grafted gut and cell culture were pretreated with 2N HCl for 30 min at 37°C and washed twice in di-sodium-tetraborat (0.1 M, pH 8.5, Merck, Darmstadt, Germany) prior BrdU immunohistochemistry. BrdU was co-stained with eGFP and various neural and glial markers (nNOS, PGP9.5 and S100β).

### Analysis of ENS cell culture and grafted gut samples

In order to determine the proportion of neuronal and glial cells generated from neurospheres *in vitro*, entire guts of four C57BL/6 mouse pups (P0) were dissected separately and ENS progenitor cells were isolated. After 7 days of proliferation, neurospheres were differentiated for another 7 days on 48 well culture plates (50 neurospheres/well) coated with Laminin, Fibronectin, Poly-L-Ornithin. Immunohistochemistry for neuronal and glial markers was performed. Ten random fields of view (20×) were captured per well and all cells positive for both, the respective marker and DAPI, were counted. In order to demonstrate that the majority of neurons develop after expansion of progenitors *in vitro*, we compared the relative number of neurons in neurospheres under proliferation versus differentiation conditions. The results from the four experiments were summarized and related to all cells staining positive for DAPI in order to determine the proportion cells positive for each of the markers.

The proportion of cells positive for the above mentioned markers was determined 12 weeks after implantation of neurospheres into the colon of adult mice. In total n = 994 (Hu c/d), n = 913 (nNOS n = 717), n = 1324 (S100beta) and n = 806 (GFAP) DAPI+/GFP+ cells were counted on 10 µm cryosections in three animals in independent preparations to determine the proportion of cells positive for the various markers. Values denote mean ± standard error of mean.

### Data analysis

Acquisition of microscopic pictures was carried out using *AxioVision* and *ZEN* Sotware provided by Carl Zeiss AG, Oberkochen. SigmaStat for Windows Version 3.5, Systat Software, Inc., Chicago, IL, USA was used for statistical analysis. Size of neurospheres was assessed in three independent experiments. Differences between groups were evaluated with Mann-Whitney Rank Sum Test. A p-value less than 0.05 was considered statistically significant.

## Results

### Generation of neurons from progenitor cells of the ENS in vitro

**Figure 1:**
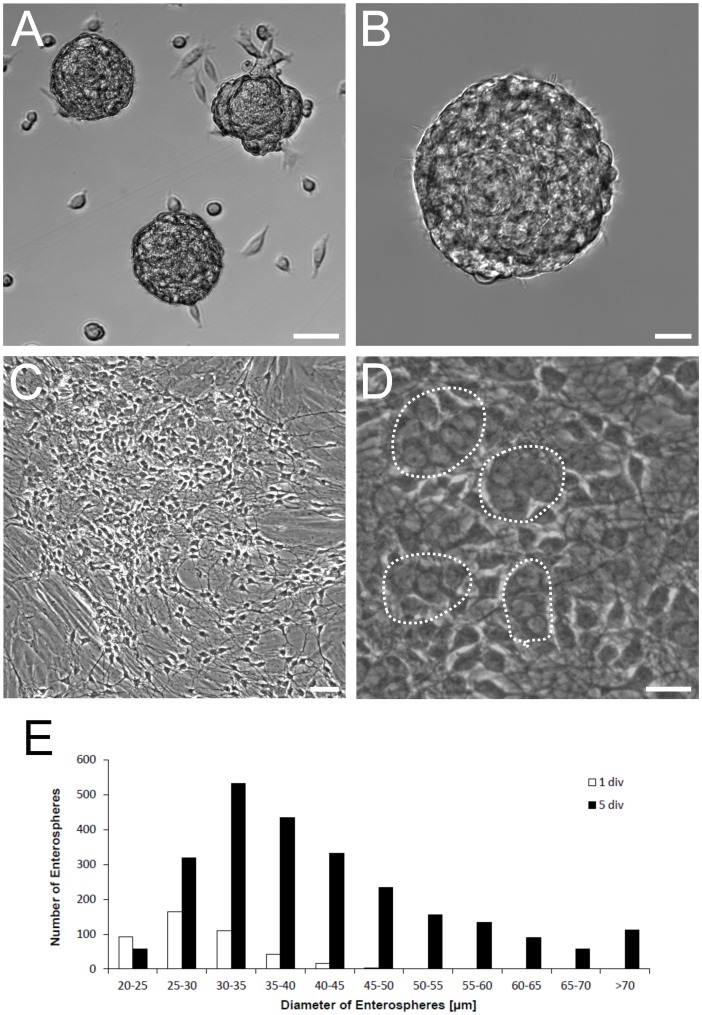
Murine ENS progenitors cultivated under proliferation conditions. A: Phase contrast live image of proliferating ENS neurospheres after 7 days in vitro. B: Higher magnification of one proliferating neurosphere. C: Phase contrast live image of ENS cells differentiated for 7 days after cell proliferation. D: Higher magnification of (C). Dotted lines circled cell groups which were later immunocytochemically proved as Hu C/D positive neurons. E: Proliferating neurospheres were classified according to their diameter into 5 µm bins. The histogram shows the number of counted neurospheres in each size class after one day (white) and 5 days (black) of culture. In total 429 and 2460 spheres were detected after 1 and 5 days, respectively. This corresponds to 90 and 518 spheres generated per neonatal small intestine at the respective time points. The diameter and number of spheres increased markedley during in vitro proliferation phase (see text). Scale bars: 50 µm in (A) and (C); 20 µm in (B) and (D).

**Figure 2:**
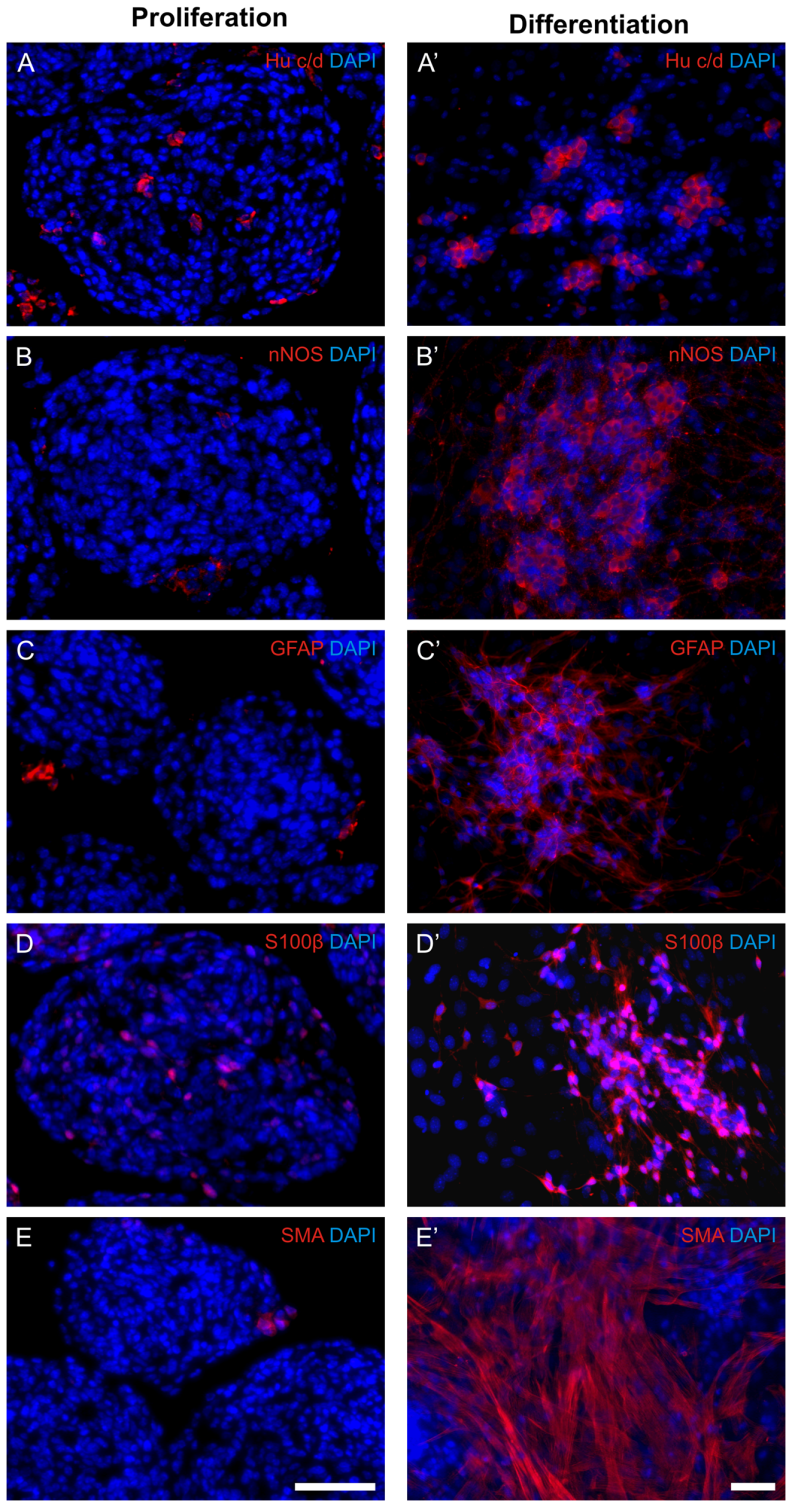
Isolated cells remain in a progenitor state during the proliferation phase. Neurospheres during proliferation (after 7 days of culture; A–E) and after another week under differentiation conditions (A′–E′). While only few cells under proliferation conditions stain positive for neuronal (A,B), glial (C,D) and muscular markers a considerable number of cells express these markers after another week of differentiation (A′–E′).

Progenitor cells of the ENS were isolated from postnatal (P0–4) mice. Cells were cultivated under serum-free conditions and supplementation of the culture medium with EGF and FGF. This resulted in formation of neurospheres (Figure 1 A, B), which were harvested after 7 days of proliferation for *in vitro* differentiation studies. To evaluate the growth of the neurospheres, we measured the size and number of spheroids larger than 20 µm in diameter after one and after 5 days *in vitro* under proliferation conditions. We thereby show a marked increase in the median diameter of neurospheres in three independent experiments (day 1: median 28.5 µm, Q25% 25.5 µm, Q75% 32.4 µm; day 5: median 38.7, Q25% 32.3 µm, Q75% 48.4 µm; Mann-Whitney Rank Sum Test, P≤0,001). Furthermore, the number of neurospheres increased during in vitro culture by the factor of 7.3±3.8 (Mean ± SD). This strongly suggests that the enlargement of spheroids was due to cell proliferation instead of fusion of neurospheres (Figure 1E). Withdrawal of growth factors and supplementation of the medium with serum resulted in adherence of the neurospheres to the culture plate, radial migration and neurite outgrowth (Figure 1 C,D; Figure 2 A′–E′). After 7 days of differentiation *in vitro* immunohistochemical analysis demonstrated cells immunoreactive for glial and neuronal markers (Figure 2 A′–E′).

### Generated neurons derive from proliferating progenitor cells of the postnatal ENS

**Figure 3:**
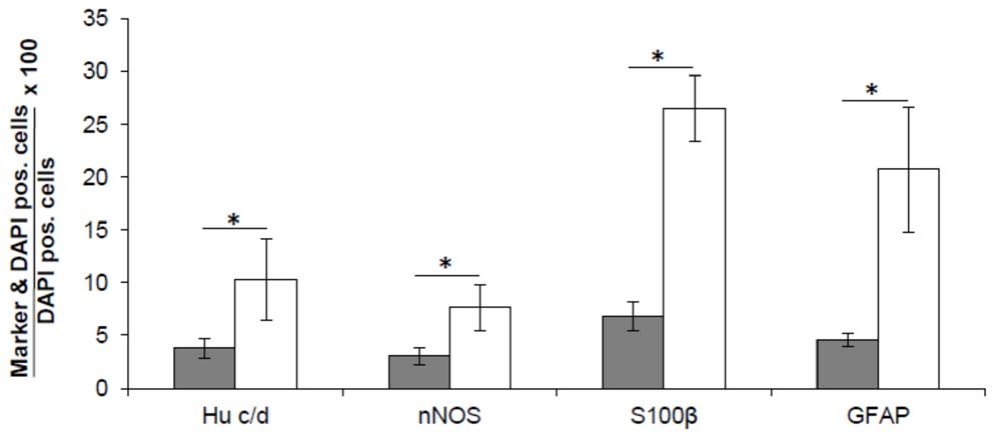
*In vitro* generated neurons and glial cells derive from isolated progenitor cells. Relative numbers of neurons (Hu c/d, nNOS) and glial cells (S100β, GFAP) were determined in neurospheres after 7 days under proliferation conditions (black) and after another 7 days under differentiation conditions (white). Data analysis demonstrated a significant increase in the relative numbers of both neuronal and glial cell types during the differentiation phase (p<0.001).

Generated neurons either derive from neurons that already exist at the time point of cell isolation or develop from progenitor cells that proliferate and differentiate while cultivated *in vitro*. In order to show that most cells during the proliferation phase remain in a progenitor state and do not express neuronal or glial markers, neurospheres were stained for the respective markers during this stage. After 1 week of proliferation *in vitro*, neurospheres contain only 3.75±0,5%, 3.02±0,5%, 6,8±1,4% and 4,5±0,7% of cells that express the neuronal markers Hu c/d and nNOS, as well as the glial makers S100β and GFAP, while 10.33±1,9%, 7.64±1,1%, 26.5±3,1% and 20.7±5,9% of cells are found to express Hu c/d, nNos, S100β and GFAP after another week of differentiation (Figure 2 and 3), respectively. This indicates that the majority of neurons and glial cells found after differentiation of neurospheres *in vitro* derived from neuronal progenitor cells. In addition a marked increase of SMA positive cells could be detected under differentiation conditions.

**Figure 4:**
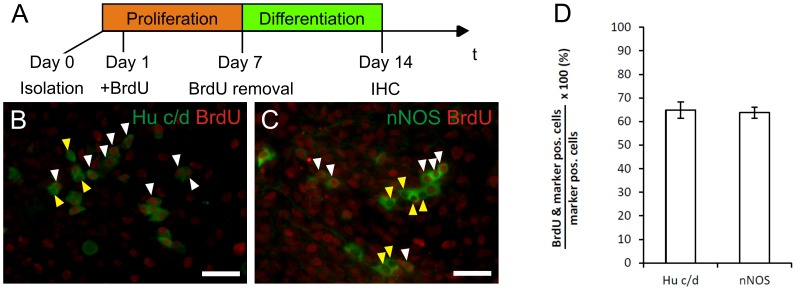
Generated neurons develop from proliferating cells *in vitro*. In order to demonstrate that neurons develop from cells that previously underwent cell division in vitro BrdU uptake studies were performed (A). 65% of Hu c/d+ and nNOS+ cells had BrdU positive nuclei (B,C; white arrow heads), while 35% of neurons did show BrdU uptake (yellow arrow heads). This provides evidence that the cells derive from expanded progenitor cells.

To underline the capacity of the progenitor cells to proliferate *in vitro*, isolated cells were incubated with BrdU during proliferation phase (Figure 4 A). When these cells were cultivated under differentiation condition for another week 65% of neurons were BrdU^+^, providing evidence that they underwent cell division *in vitro* prior differentiation into neurons (Figure 4 B,C).

Long-term cultivation of postnatal ENS progenitor cells could be demonstrated by generating tertiary enteric neurospheres. Exposure of proliferating tertiary spheres to BrdU 26 days after cell isolation and subsequent differentiation resulted in cells staining positive for both neuronal markers (TuJ1 or Hu c/d) and BrdU, showing that a proportion of cells maintain their proliferative and differentiation potential after 4 weeks *in vitro* (Figure S1).

### Proliferated postnatal enteric neurons express voltage gated sodium channels

**Figure 5:**
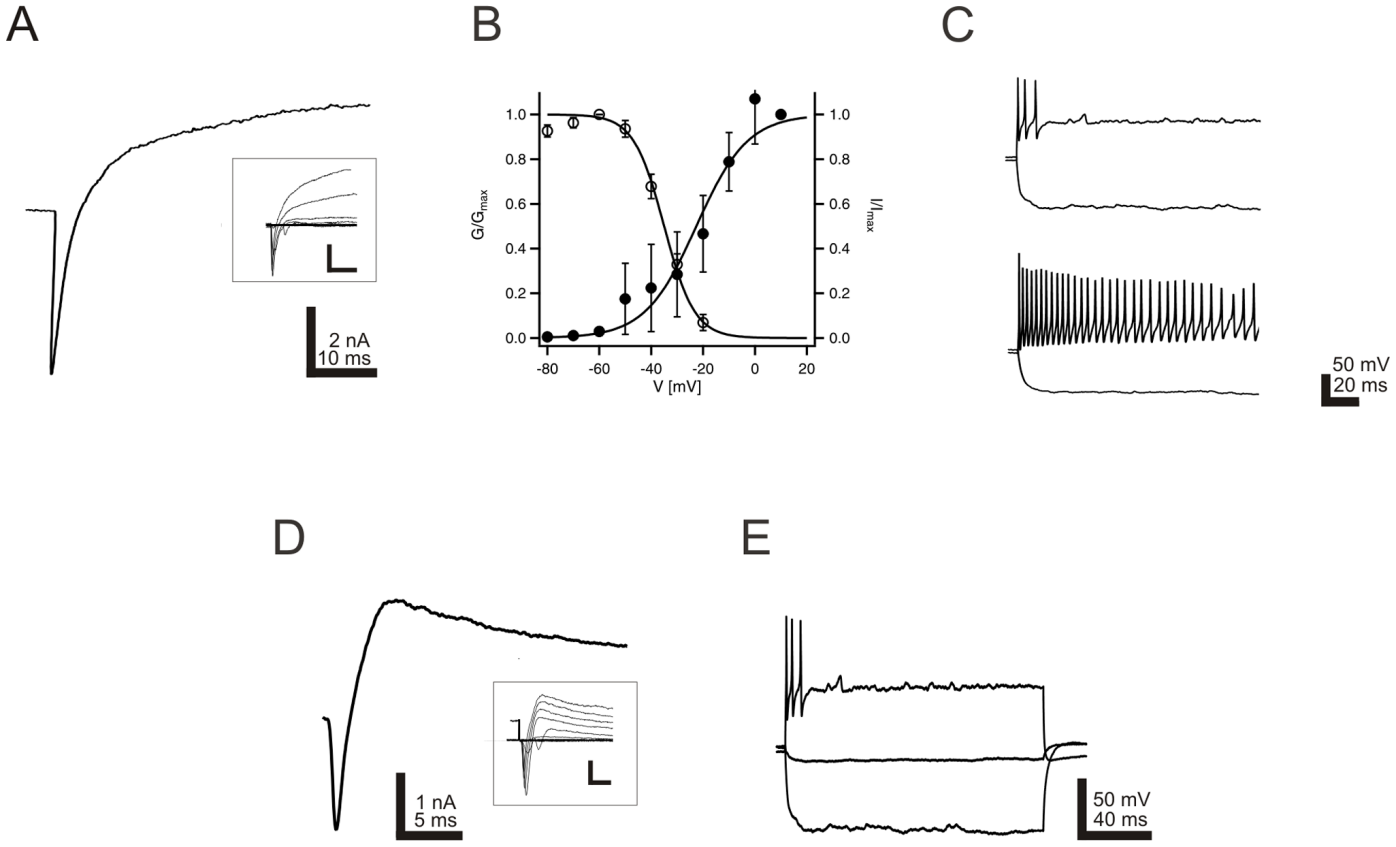
*In vitro* generated neurons display defined electrophysiological properties. Electrophysiological properties of neurons derived from postnatal murine neurospheres. A: Exemplary voltage-gated currents elicited by a depolarizing pulse from −120 mV to +10 mV. Inserts display superpositions of consecutive current responses obtained by pulses from −120 to +30 mV in 10 mV increments. Marked transient inward sodium currents are superseded by outward potassium currents. B: Activation (filled circles) and inactivation (open circles) curves of voltage gated sodium currents. Conductance values were calculated from the respective sodium peak current amplitudes and normalized to the conductance at +10 mV. Data could be best described using a Boltzmann equation (data from 14 cells). Error bars indicate SEM. C: Firing patterns could be separated in two main groups of neurons, namely phasic (upper) and tonic (lower) patterns. D: Representative current response of a neuron, which postexperimentally proved to stain positive for biocytin and BrdU, to a depolarizing pulse from −120 to +10 mV. A prominent inward sodium current is visible at the beginning of the pulse, followed by a longer lasting potassium current. Inserts display superpositions of consecutive current responses obtained by pulses from −120 to +30 mVin 10 mV increments. E: The same neuron was capable to generate action potentials when depolarized.

In order to investigate the electrophysiological properties of cells differentiated from postnatal enteric neurospheres, voltage and current clamp experiments were performed *in vitro*. 22% of examined cells (29 out of 130) exhibited voltage-activated sodium currents (Figure 5 A), which could be blocked by the specific sodium blocker tetrodotoxin (not shown). Voltage dependence of sodium channel activation and steady-state inactivation was studied using test pulses (−80 to +20 mV) and prepulses (−120 to −30 mV) to the membrane potential, respectively. For activation the mid-point potential and slope factor was −22.6±1.7 mV and 9.9±1.5, for inactivation −35.0±2.6 mV and 15.1±1.6, respectively (n = 29; Figure 5 B). In 50% of investigated neurosphere derived cells action potentials could be elicited. Phasic and tonic firing patterns could be discriminated (Figure 5 C), as described previously for adult enteric myenteric neurons of the guinea pig (Vogalis, Hillsley, & Smith, 2000).

**Figure 6:**
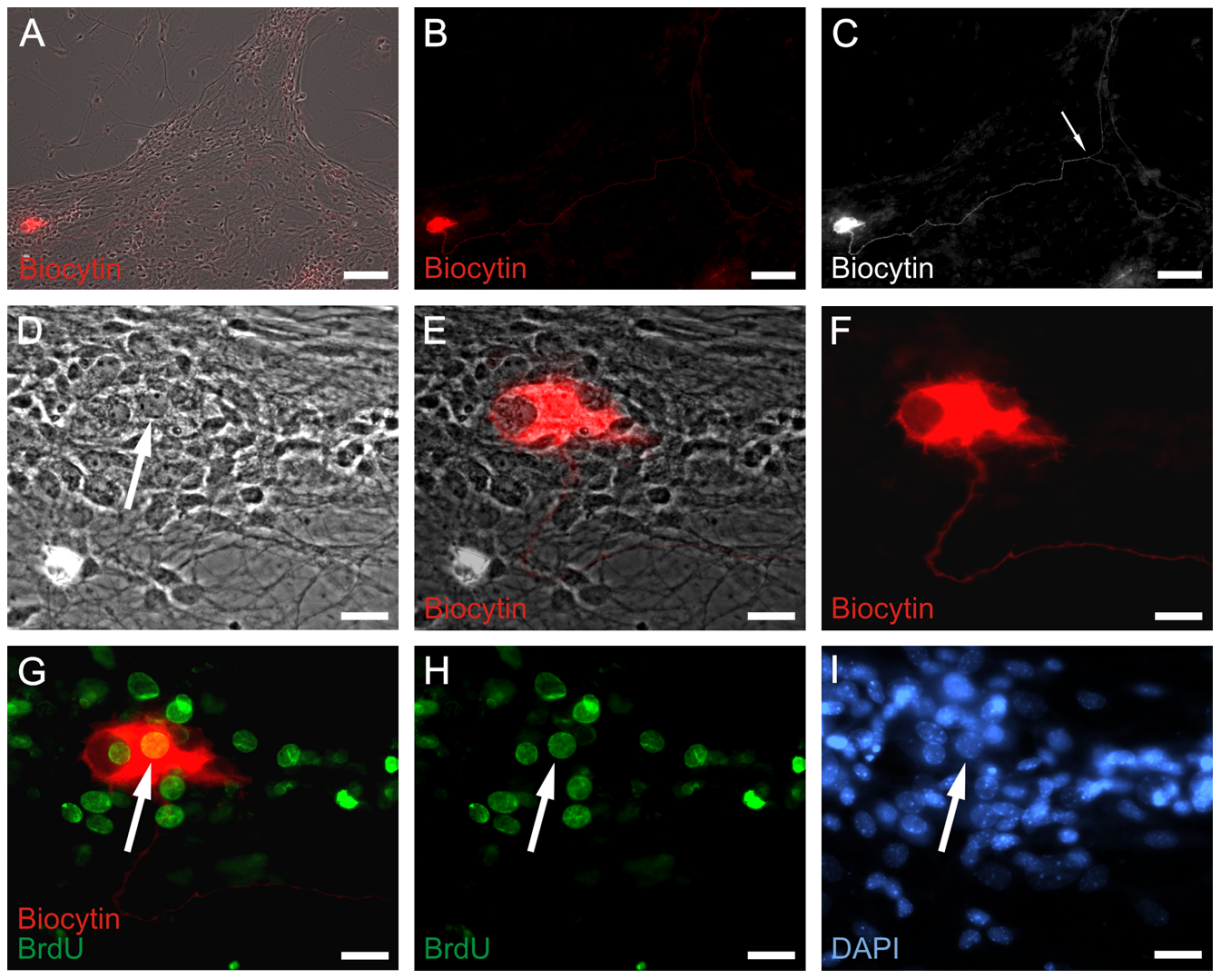
Electrophysiologically active neurons derive from proliferating progenitors *in vitro*. Sodium channel positive neuron was derived from a proliferated ENS progenitor cell in vitro. The ENS cells were proliferated for 7 days and differentiated for 18 days prior to patch clamp recording. Under proliferation culture condition, progenitors were incubated with BrdU for 6 days. During patch clamp measurement, the recorded cell was filled with biocytin. A: Overview of combined bright-field/biocytin fluorescence (red) view of differentiated ENS cells. B: Biocytin fluorescent staining of (A). C: Enhanced black/white view of (B). The length between perikaryon and the junction of bifurcation (arrow) is 665 µm. D: Higher magnification of bright-field view shown in (A). E: Higher magnification of (A). F: Higher magnification of (B). G: Combined immunocytochemical staining of biocytin (red) and BrdU (green). H: BrdU immunofluorescence of (G). I: Nuclei staining with DAPI (blue) of the same area shown in (D–H). Arrows in (D), (G), (H) and (I) indicate the same nucleus of patched and biocytin filled neuron. Scale bars: 100 µm in (A–C); 20 µm in (D–I).

In oder to demonstrate that a subset of the examined neurons derive from expanded progenitor cells, the culture was exposed to BrdU during the proliferation phase. During electrophysiological characterization cells were filled with biocytin. This procedure allowed us to conclude postexperimentally, if the recorded cell had proliferated *in vitro*. Figure 6 demonstrates a BrdU and biocytin costained neuron that previously exhibited voltage-activated sodium currents. A representative current response of a neuron, which proved to stain positive for biocytin and BrdU is shown in Figure 5 D and E. This provides evidence that ENS progenitor cells proliferate in vitro and generate electrophysiologically and morphologically defined neruons *in vitro*.

### Implantation of progenitor cells of the postnatal ENS into the colon of adult mice

**Figure 7:**
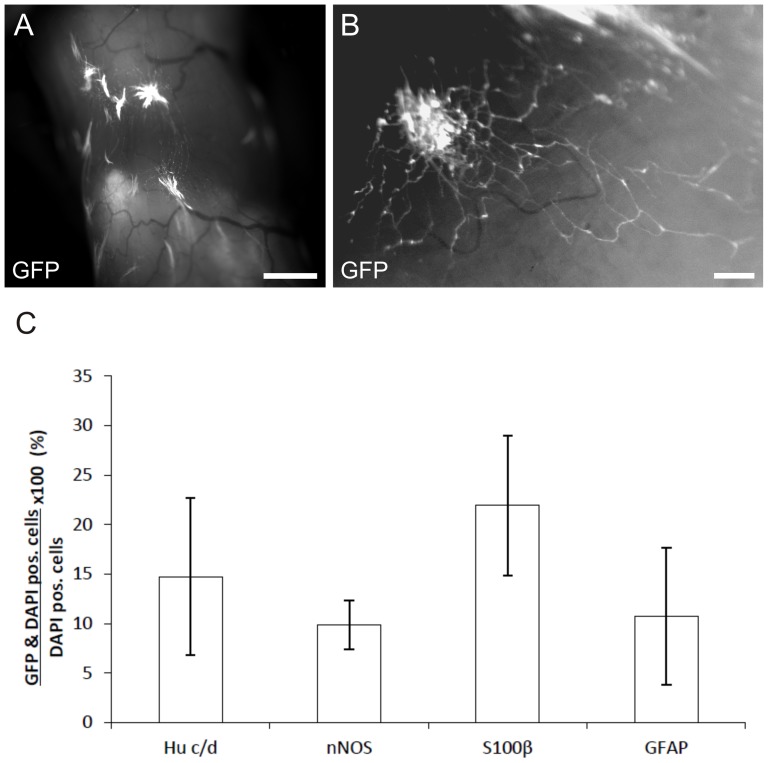
Generated neurospheres colonize murine colonic bowel wall *in vivo*. Combined fluorescence and bright field microscopy of native explanted murine distal colon twelve weeks after implantation of eGFP expressing neurosphere like bodies generated from postnatal (P0) progenitor cells of the ENS. A: Engraftment of multiple neurospheres into the distal colonic wall. Colonization of the adjacent bowel tissue by cell migration and sprouting can be observed over several milimeters. B: Higher magnification of migrating eGFP expressing cells with extentions within the bowel wall. C: Relative proportion of implanted cells positive for various neural and glial markers in relation to all implanted cells. Scale bar: 1 mm (A) and 100 µm (B).

Neurospheres generated from postnatal murine gut of eGFP transgenic mice were injected into the distal colonic bowel wall of NOD.Cg-Prkdc^scid^ IL2rg^tm1WJl^ mice. Recipient mice developed normally compared to their non-operated littermates and did not present signs of constipation. Implanted cells were identified by eGFP expression in 13 of 16 mice and could be detected up to 12 weeks postoperatively (end of study). Gross examination at the time-point of dissection of the recipient gut did not show obvious changes in the caliber at the site of implantation. Upon injection of spheres radial migration of cells and sprouting of cell extensions into the adjacent tissue was observed (Figure 7 A and B). However, no directed migration towards the myenteric or submucosal plexus could be demonstrated. In addition, inter-individual variation in cell delivery was evident, localizing injected cells within various areas of the bowel wall, ranging from the submucosal to the subserosal layer.

**Figure 8:**
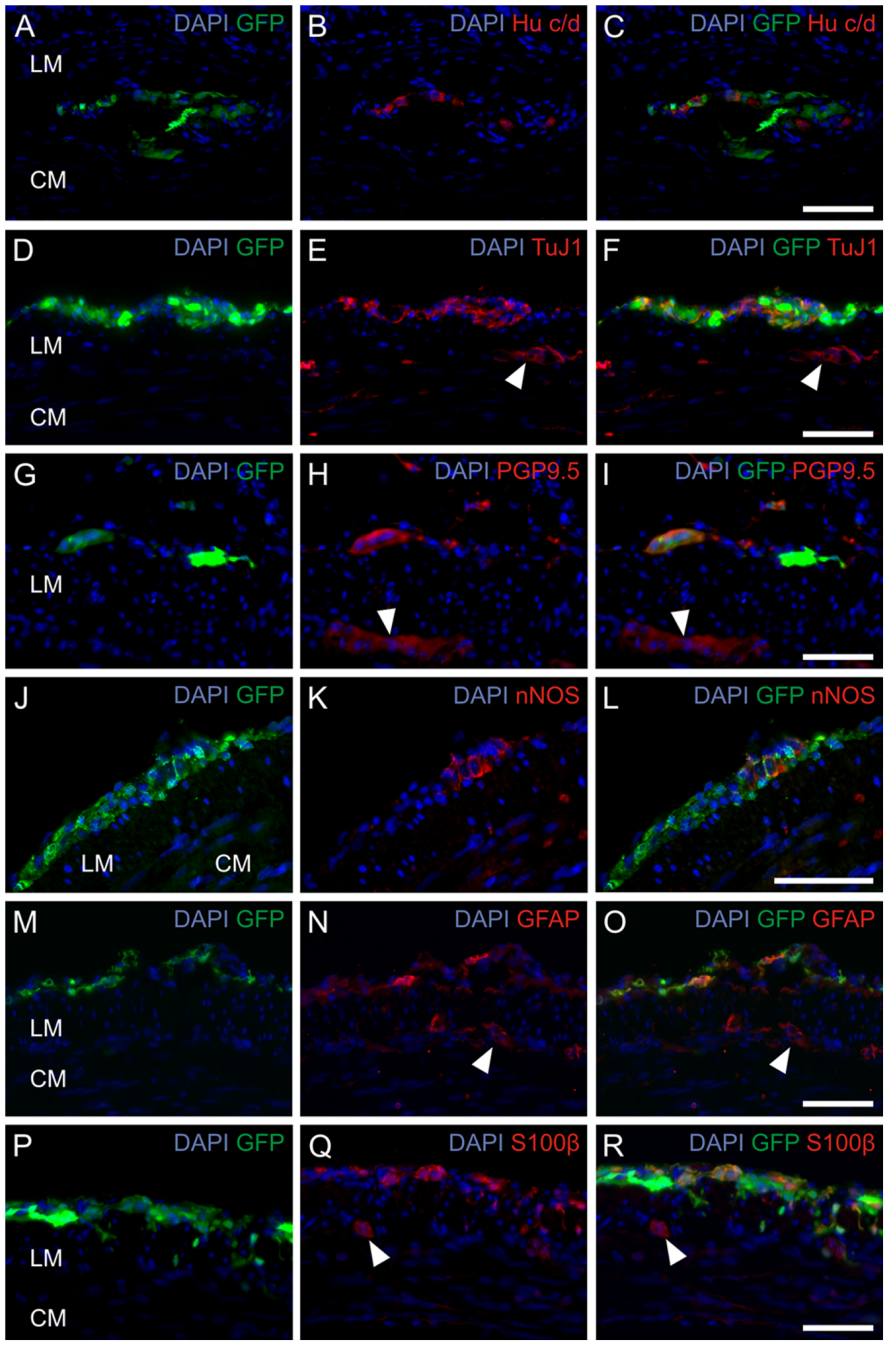
*In vivo* implanted progenitor cells differentiate into neurons and glial cells. Immunohistochemical analysis of postnatal progenitor cells of the ENS 12 weeks after implantation into the distal colon of NOD.CB17-*Prkdc^scid^*/NcrCrl mice. Staining for the neural makers Hu c/d (A–C), TuJ1 (D–F), PGP9.5 (G–I), nNOS (J–L), GFAP (M–O) and S100β (P–R). The cells could be located within the region of the myenteric plexus (A–C) or within the longitudinal muscle layers (D–L). Ganglia of the recipient mouse are marked by white arrowheads. Nuclei were DAPI stained (blue). Scale bar: 50 µm.

Immunohistochemical analysis of implanted cells 12 weeks after implantation demonstrated the presence of cells immunoreactive for various neuronal and glial markers (Figure 8). Immunoreactivity for the pan-neuronal marker Hu c/d was identified in 14.7±8.0% of implanted cells. Positive staining for nNOS could be detected in 9.8±2.5% as well as immunoreactivity for S100β and GFAP in 21.9±7.1% and 10.7±6.9% in relation to all counted cells, respectively (Figure 7 C).

**Figure 9:**
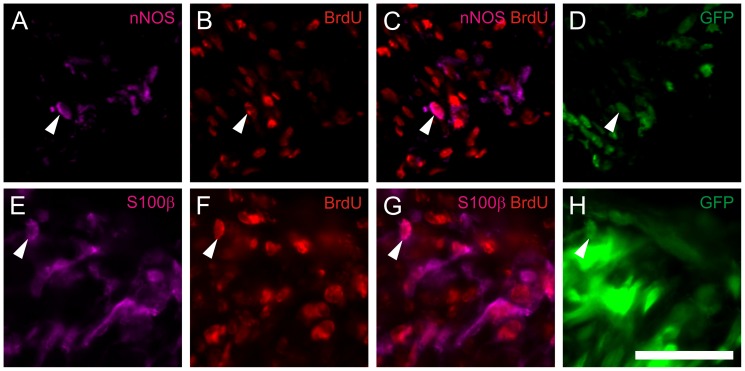
*In vivo* generated neurons derive from *in vitro* expanded progenitor cells. BrdU loaded postnatal progenitor cells 3 weeks after implantation into the distal colon of NOD.CB17-*Prkdc^scid^*/NcrCrl mice. The arrows indicate triple-staining of injected cells for eGFP, BrdU and various markers as nNOS (A–D), and S100β (E–H). Triple staining provides evidence that the cells stained positive for the neural and glial markers underwent cell division and incorporated BrdU during the proliferation phase *in vitro* prior implantation into the mouse colon. The BrdU staining remains restricted to implanted eGFP positive cells. Cell nuclei are stained with DAPI (blue). Scale bar: 50 µm.

In additon, neurospheres were loaded with BrdU. 3 weeks after implantation into the mouse colon, multiple staining for eGFP, neural markers, and for BrdU was performed on cryosections. Immunohistochemical analysis demonstrated implanted eGFP positive cells co-staining with BrdU, including cells immunoreactive for nNOS and S100β. This indicates that in vivo generated neurons and glial cells derive from progenitor cells that have proliferated *in vitro* (Figure 9).

## Discussion

Increasing evidence has accumulated that progenitor cells originating from postnatal gut represent an adequate cell source to regenerate the ENS [16]–[18]. Several studies indicated long-term propagation of neurospheres generated from cells of the postnatal rodent ENS [19]–[22]. To further characterize the cells' properties in more detail we generated enteric neurospheres from postnatal mice. Isolated cells showed a multipotent behavior and generated neurons and glial cells when cultivated under differentiation culture conditions. After one week of proliferation and another week under differentiation conditions, about 10% of the cells were immunoreactive for the pan-neuronal marker Hu c/d, comprising 7.6% of cells staining for nNOS. These cells mainly derive from progenitor cells that proliferated during cultivation in vitro as demonstrated by BrdU uptake studies. The fact, that only few cells express neuronal or glial markers under proliferation conditions underlines the progenitor characteristics of the cultivated cells. In addition demonstration of a significant increase of size and number of neurospheres during *in vitro* culture, as well as the cells' capacity to maintain their long-term proliferation and differentiation potential by generating tertiary enteric neurospheres (Figure S1) indicates the potential of the cells to generate an adequate cell pool for transplantation experiments.

Electrophysiological studies on cells isolated from developing and postnatal bowel tissue provided valuable information about the electrical functionality of enteric neurons [34]–[37]. However, only limited data exist about the capability of postnatal progenitors of the ENS to generate cells with distinct neuronal electrophysiological properties [26]. In the present study, we were able to demonstrate the electric activity of neurons that were generated from postnatal murine bowel tissue by patch-clamp studies. Voltage-gated sodium channels could be detected in cells derived from postnatal murine neurospheres. In addition, characteristic sodium channel gating and the capability to fire action potentials underline the neuronal phenotype of the generated cells.

In order to demonstrate that electrophysiologically recorded neurons derived from expanded ENS progenitors BrdU-uptake experiments were performed. We are able to provide direct evidence by co-labeling experiments that neurons with distinct electrophysiological properties had differentiated from expanded progenitors of the postnatal ENS. The capability of cells of embryonic origin to give rise to neuronal and glial cells, when implanted into both ganglionated and aganglionic postnatal bowel *in vitro* and *in vivo* could be demonstrated by different groups [38]–[42]. In addition, improvement of bowel motility was observed upon implantation of embryonic progenitors or cell lines of the ENS in various animal models [42]–[44]. However, taking ethical and practical aspects into account, a cell based therapeutic approach utilizing postnatal and easy accessible tissue as cell source is desirable. Although previous reports indicate the potential of postnatal progenitor cells of the ENS to integrate into adult gut of rats, these studies were not able to either demonstrate long-term survival of implanted cells or to provide direct evidence that the implanted cells derive from ENS precursors that proliferated in vitro prior implantation [29]–[30]. In a recent study Hotta et al. [32] demonstrated the potential of postnatal progenitor cells of the ENS, isolated form *Ednrb^Kik^* mice [32] to colonize the distal colon of newborn C57BL/6 mice. In *Ednrb^Kik^* mice progenitor cells of the ENS express the fluorophor Kikume, which allows straight forward isolation of the cells by flourescence activated cell sorting. In contrast to this study, we investigated a cell population that was isolated by permissive culture conditions and spontaneous neurosphere formation only. Isolation of ENS precursor cells from postnatal murine gut and implantation of the cells into the colon of immunocompromised mice provided a robust experimental setting. We were able to study the long-term behavior of transplanted postnatal ENS precursor cells within the microenvironment of the normally ganglionated adult gut, mimicking the clinical scenario of autologous progenitor therapy. 12 weeks after implantation (end of study) histochemical analysis demonstrated that grafted cells survived and migrated within the gut wall, confirming the results of the previous work of Hotta et al. [32] who demonstrated survival an migration of postnatal progenitor cells of the ENS for 4 weeks, when implanted into the neonatal mouse gut.

Implanted cells could be found either in the subserosal, muscular or submucosal layer of the bowel. However, the cells did not show directed migration towards the region of the myenteric plexus. This is in contrast to other studies, which reported directed migration and engraftment of embryonic ENS precursor cells [38], [41] into the myenteric plexus, even when injected intraperitoneally into neonatal HSCR mouse models. Both the embryonic origin and the young age of the recipients might have provided guidance cues in the reported studies, which might not be present anymore in adult mice receiving ENS progenitors isolated from postnatal gut. Therefore it remains to be investigated, whether the postnatal nature of the ENS progenitor cells, or the normal anatomy and age of recipient animals are responsible for the limited directed migration in the reported study. Genetic modification of donor cells, improvement of cell delivery techniques or alteration of the host tissue might be necessary to overcome this problem.

Immunohistochemistry for Hu c/d, nNOS, S100β and GFAP emphasized neuronal and glial differentiation of implanted cells. This indicates the permissive microenvironment of the normally ganglionated bowel for differentiation of neural progenitor cells of the ENS isolated from postnatal gut.

BrdU labeling of ENS progenitors *in vitro* proved that implanted and differentiated cells in vivo derived from expanded progenitor cells. This is an important issue, since in vitro expansion of the progenitor cell pool is supposed to be one of the major requirements to generate a sufficient number of neural progenitor cells to colonize a defective or aganglionic bowel segment.

Our experiments indicate the capability of postnatal progenitor cells of the ENS to proliferate and to give rise to neurons and glial cells *in vitro*. The combination of BrdU uptake studies with electrophysiological analysis provide direct evidence that generated functional neurons derived from proliferated progenitors *in vitro*. In addition, the cells proved to survive, migrate and differentiate into neurons upon implantation into ganglionated bowel *in vivo*. The presented data do not provide evidence that the implanted cells either positively or negatively influence bowel motility. However the study provides the basis for further experiments that are necessary to prove that postnatal progenitor cells of the ENS implanted into the postnatal bowel wall connect to the smooth muscle, mucosa and recipient ENS and generate neural circuits that are able to coordinately improve motility in enteric neuropathies.

## Supporting Information

Figure S1 **Neurons generated from tertiary enteric neurospheres after 4 weeks of proliferation and 7 days under differentiation conditions** ***in vitro*** **.** The neurospheres were exposed to BrdU after 25 days of proliferation (G). Immunohistochemistry shows cells positive for BrdU and neuronal markers (nNOS, A–C and Hu c/d, D–F), demonstrating that the cells maintain their proliferative and differentiation potential even after 4 weeks under proliferation conditions in vitro.
(TIF)
