# Supplementary figures and images for: Isolation, Expansion and Transplantation of Postnatal Murine Progenitor Cells of the Enteric Nervous System
Source: PLoS One. 2014 May 28;9(5):e97792. doi: 10.1371/journal.pone.0097792 (PMC4037209; doi:10.1371/journal.pone.0097792)

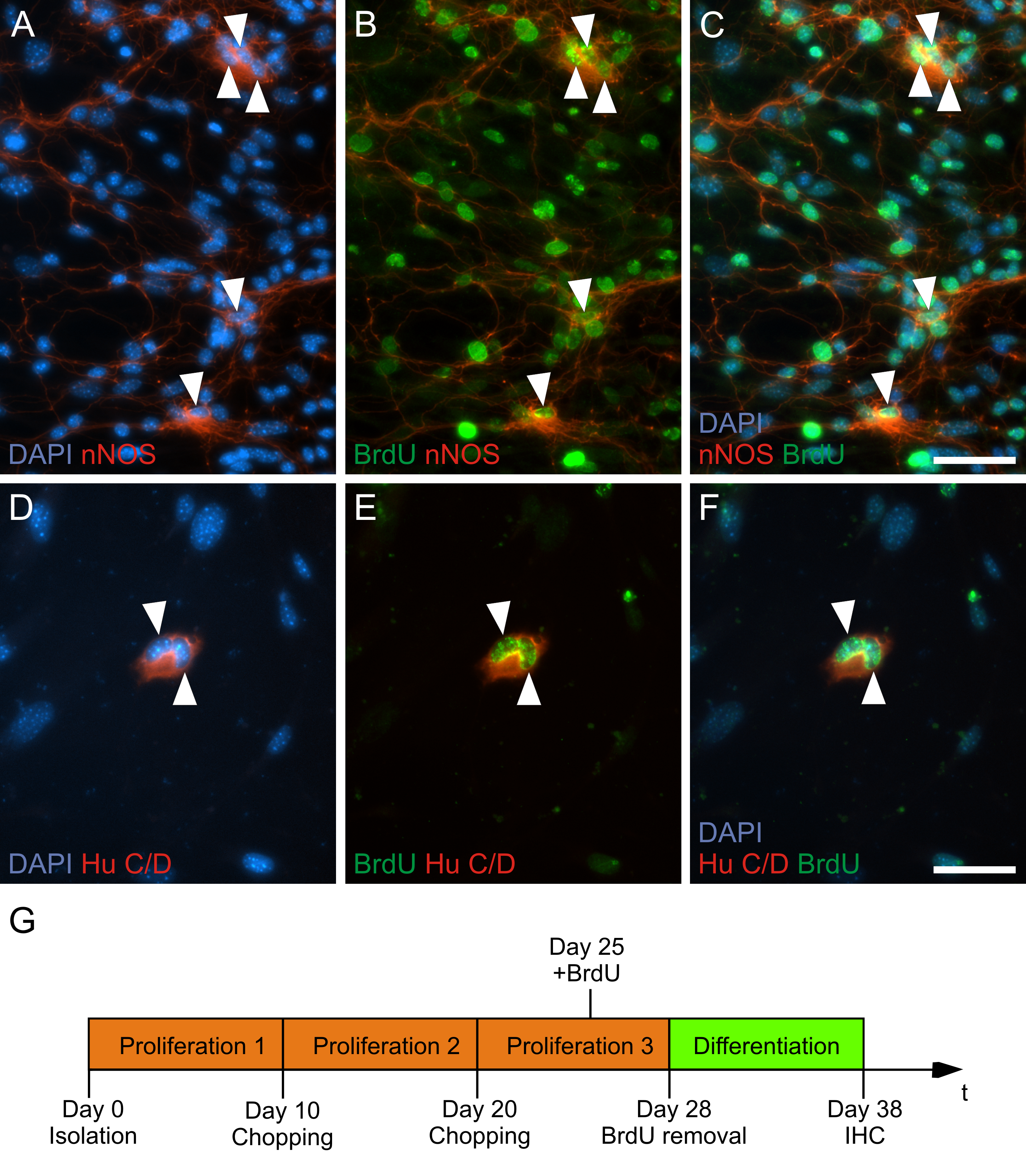

Supplement: Figure S1 — Neurons generated from tertiary enteric neurospheres after 4 weeks of proliferation and 7 days under differentiation conditions in vitro . The neurospheres were exposed to BrdU after 25 days of proliferation (G). Immunohistochemistry shows cells positive for BrdU and neuronal markers (nNOS, A–C and Hu c/d, D–F), demonstrating that the cells maintain their proliferative and differentiation potential even after 4 weeks under proliferation conditions in vitro. (TIF) [file pone.0097792.s001.tif]
